# Equity in newborn care, evidence from national surveys in low- and middle-income countries

**DOI:** 10.1186/s12939-021-01452-z

**Published:** 2021-06-05

**Authors:** Kimberly Peven, Lindsay Mallick, Cath Taylor, Debra Bick, Louise T. Day, Lionel Kadzem, Edward Purssell

**Affiliations:** 1 Florence Nightingale Faculty of Nursing, Midwifery & Palliative Care, King’s College London, London, UK; 2 Maternal and Newborn Health Group, London School of Hygiene & Tropical Medicine, London, UK; 3 University of Maryland, College Park, MD USA; 4 Avenir Health, Glastonbury, CT USA; 5 School of Health Sciences, University of Surrey, Guildford, UK; 6 Warwick Clinical Trials Unit, University of Warwick, Coventry, UK; 7 Ministry of Health, Yaoundé, Cameroon; 8 School of Health Sciences, City, University of London, London, UK

**Keywords:** Infant, Newborn, Health equity, Socioeconomic factors, Postnatal care

## Abstract

**Background:**

High coverage of care is essential to improving newborn survival; however, gaps exist in access to timely and appropriate newborn care between and within countries. In high mortality burden settings, health inequities due to social and economic factors may also impact on newborn outcomes. This study aimed to examine equity in co-coverage of newborn care interventions in low- and low middle-income countries in sub-Saharan Africa and South Asia.

**Methods:**

We analysed secondary data from recent Demographic and Health Surveys in 16 countries. We created a co-coverage index of five newborn care interventions. We examined differences in coverage and co-coverage of newborn care interventions by country, place of birth, and wealth quintile. Using multilevel logistic regression, we examined the association between high co-coverage of newborn care (4 or 5 interventions) and social determinants of health.

**Results:**

Coverage and co-coverage of newborn care showed large between- and within-country gaps for home and facility births, with important inequities based on individual, family, contextual, and structural factors. Wealth-based inequities were smaller amongst facility births compared to non-facility births.

**Conclusion:**

This analysis underlines the importance of facility birth for improved and more equitable newborn care. Shifting births to facilities, improving facility-based care, and community-based or pro-poor interventions are important to mitigate wealth-based inequities in newborn care, particularly in countries with large differences between the poorest and richest families and in countries with very low coverage of care.

**Supplementary Information:**

The online version contains supplementary material available at 10.1186/s12939-021-01452-z.

## Introduction

High coverage of newborn care is essential to improving newborn survival and meeting global sustainable development goals (SDG) [1]. However, large gaps exist in access to quality care and health outcomes between and within countries [2]. Reducing the equity gap in access to care is needed to reach newborns with the greatest need, not only in relation to wealth-based inequity but other forms of social marginalisation [1]. The World Health Organisation (WHO) has outlined a vision where, ‘every pregnant woman and newborn receives quality care throughout pregnancy, childbirth and the postnatal period,’ describing equitable care as a key component of quality care [3].

Access to skilled care at birth, but also improved quality of newborn care provided by the skilled attendant is associated with improved neonatal survival. In sub-Saharan Africa, where coverage of skilled care at birth in sub-Saharan Africa ranges from 29% in Niger to 93% in Congo, newborns with a skilled attendant at birth were 16% less likely to die in the first 2–27 days of life, compared to those born without a skilled attendant. Furthermore, where a skilled attendant was present a birth, newborns breastfed within a short time of being born and those who were weighed at birth were significantly less likely to die in the first 2 days of life [4]. While the need for skilled care around the time of birth is universal, poverty and distance to facilities are important determinants of hospital birth [5].

Service readiness in healthcare facilities, measures capacity to provide care including trained personnel and infrastructure, equipment, and commodities [6], which varies greatly between countries and facilities [7]. De Graft-Johnson et al. (2017), in a cross-sectional study of facility-based births in six countries found that even availability of basic supplies for newborn care was variable. For example, availability of towels and blankets to promote thermo-regulation of newborns ranged from 8 to 53% of facilities, and availability of cord ties or clamps ranged from 36 to 99.5% [8]. Access to health facilities with higher service readiness is associated with improved newborn care practices [9]. Important differences in service readiness have been shown between urban and rural facilities as well as between public and private facilities, suggesting inequitable access to high quality newborn care [10].

Inequities persist despite increases in coverage of essential maternal and newborn care interventions [11], with considerable evidence that social determinants influence facility birth [12]. Inequities may result from discrimination or differential treatment at the point of care or from delaying care due to fear of discrimination [13]. Inequities also result from other barriers to accessing care or quality care such as direct and indirect costs, influence of others on decision making, and transportation and access [14]. Wealth-based inequities have been shown in access to maternal, newborn, and child health interventions, particularly for presence of a skilled attendant at birth [15]. Additionally, structural factors such as women’s empowerment or decision making power is also associated with use of maternal health services, including skilled delivery services [16]. We adapted a conceptual framework from the United Nations Development Programme on the social determinates of maternal health [17] to explore individual, family, context, and structural inequities in access to quality newborn care.

### Study aims

This study aims to describe equity in reported receipt of newborn care interventions in the first 2 days of life, specifically:

1. Describe coverage and co-coverage of newborn care interventions by wealth quintile for newborns born in facilities and at home.
2. Determine associations between social determinants of health and reported receipt of appropriate newborn care.

## Methods

### Data

The Demographic and Health Survey Program (DHS) collects health data in high burden mortality settings including newborn care. Surveys are completed at household- and individual-levels, focusing on report from women of reproductive age (15–49 years). Complex, multi-stage sampling and stratification produce nationally-representative results for each country [18].

### Population

We analysed secondary DHS survey data since 2015 from low- and low middle-income countries in sub-Saharan Africa and south Asia which included questions on newborn care interventions in the first 2 days of life. We included last (most recent) live births in the 2 years before the survey. Exclusion criteria were births in the 2 days before the survey and neonates who died before the second day. Table 1 shows the included countries, survey years, and number of women interviewed.

**Table 1:** List of included countries from DHS, survey year, and sample sizes

Country	Survey year	Number of women interviewed^a^	Number of facility births in the 2 years before the survey^b^	Number of non-facility births in the 2 years before the survey^b^
**Angola**	2015–16	14,379	2492	2785
**Benin**	2017–18	15,928	4584	806
**Burundi**	2016–17	17,269	4576	782
**Cameroon**	2018	13,527	2605	1238
**Ethiopia**	2016	15,683	1521	2701
**Guinea**	2018	10,874	1607	1341
**Malawi**	2015–16	24,562	6112	455
**Mali**	2018	10,519	2846	1229
**Nepal**	2016	12,862	1259	699
**Nigeria**	2018	41,821	5095	7522
**Pakistan**	2017–18	12,264	2749	1106
**Senegal**	2017	16,787	3531	870
**Tanzania**	2015–16	13,266	2656	1435
**Uganda**	2016	18,506	4421	1360
**Zambia**	2018	13,683	3320	525
**Zimbabwe**	2015	9955	1961	456

^a^Weighted, from ICF International [19]

^b^ includes only most recent live-born children surviving the first 2 days of life

### Analysis

#### Outcomes

We created a co-coverage index of provider-initiated early newborn care interventions, using a method similar to Victora et al. [20] and Carvajal-Aguirre et al. [9]. We included five provider-initiated interventions included in the WHO standards for maternal and newborn care [21]: 1) examining the umbilical cord, 2) taking the newborn’s temperature, 3) counselling on danger signs in the newborn, 4) counselling on breastfeeding, and 5) observing breastfeeding (Table 2). The primary outcome measure was receipt of 4–5 of these interventions provided in the first two days of life and hereafter called “appropriate newborn care”.

**Table 2:** Newborn care intervention survey questions. Newborn care interventions and question wordings from the phase seven DHS model questionnaire [22]

Intervention	Question
**Umbilical cord check**	457 a) During the first two days after (NAME)‘s birth, did any health care provider do the following: Examine the cord?
**Temperature measurement**	457 b) During the first two days after (NAME)‘s birth, did any health care provider do the following: Measure (NAME)‘s temperature?
**Danger sign counselling**	457 c) During the first two days after (NAME)‘s birth, did any health care provider do the following: Counsel you on danger signs for newborns?
**Breastfeeding counselling**	457 d) During the first two days after (NAME)‘s birth, did any health care provider do the following: Counsel you on breastfeeding?
**Breastfeeding observation**	457 e) During the first two days after (NAME)‘s birth, did any health care provider do the following: Observe (NAME) breastfeeding?

#### Key independent variables

**Fig. 1:**
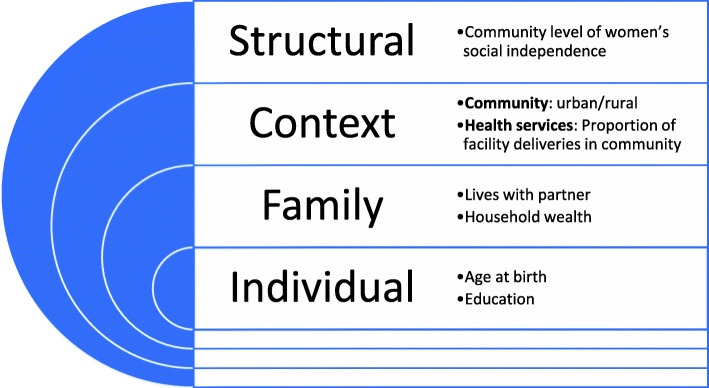
Conceptual framework for social determinants of health, adapted from the United Nations Development Programme [17]

Key predictor variables focused on social determinants of health using an adapted person-centred conceptual framework (Fig. 1) where individual women and their newborns sit at the centre, encircled by their families and the wider community and structural contexts [17].

1. Individual level factors
  - Age at birth – categorical (< 20, 20–34, 35+ years) – age is a known determinant of facility birth [23]
  - Education level – binary (no education or primary / secondary education and higher) – duration of education is associated with postnatal care use [24]
2. Family level factors
  - Lives with partner – binary (lives with / does not live with partner) – family members influence women’s choices [17]
  - Household wealth quintile – categorical (poorest / poorer / middle / richer / richest) – limited financial resources may constrain access to services via ability to pay fees or transport [17]
3. Contextual factors
  - Community: urban/rural residence – binary (urban / rural) – urban residence and distance to health facilities is associated with postnatal care contacts [24]
  - Health services: community-level facility birth rate – categorical (proportion of women in the survey cluster who had a birth in the last 5 years who gave birth in a health facility calculated at the cluster level and applied to all women in the cluster, ranked in terciles by country) – proxy for availability of birth/newborn services
4. Structural factors
  - Community level of women’s social independence – categorical (calculated and averaged across all partnered women in each survey cluster using the Survey-based Women’s emPowERment index (SWPER) [25] and applied to all women in the cluster, ranked in terciles by country). The social independence index is a weighted summary of 15 items, with weights determined by loadings from a principal component analysis. The social independence index is weighted most heavily by items including frequency of reading newspaper or magazines, education, age at first birth, and the difference between a woman and her partner in terms of age and education. Women’s status in society is an important social determinant of maternal health [17]

### Statistical analysis

Simple weighted descriptive statistics on individual intervention coverage and co-coverage by birth location, wealth quintile, and country were calculated. We visually examined patterns of wealth-based inequities (Victora et al. [20]) assigning “top inequity,” “bottom inequity,” and “linear inequity”. Top inequity, also described as ‘mass deprivation’ [26], is when the majority of the population are deprived and only a minority have access to care. Bottom inequity, also described as ‘marginalisation’ [26], is when the majority of the population have access to care but a minority are excluded. Linear inequity, also described as queuing, lies somewhere between top and bottom inequity, with a linear relationship between wealth and access [26].

In descriptive analyses, individual-level weights were applied to account for sampling probability and non-response to ensure each sample was nationally representative. Descriptive results are presented for facility birth and home birth separately.

Multilevel, multivariable logistic regression models were fitted, by country for facility birth only to assess the association between the factors in the conceptual framework and appropriate newborn care. For the multilevel models, individual-level weights were denormalised and cluster-level weights were approximated with equal allocation between individual and cluster levels (α = 0.5) using a method described by Elkasabi et al. [27]. The models were adjusted for the independent variables listed above.

All statistical analyses for this study were conducted in R [28] and Stata, using the survey package [29] in R and applying the *svy* commands in Stata where appropriate to adjust for the complex sampling design.

## Results

### Sample characteristics

Table 3 shows background characteristics of the population, by birth location. Age distribution was generally older amongst home births. The proportion of home births where women were 35 years or older at the time of birth ranged from 5.1% in Nepal to 24.7 % in Burundi. Amongst facility births, secondary or higher education ranged from 13.4% in Burundi to 72.2% in Zimbabwe. Amongst home births this ranged from 3.0% in Benin to 42.9% in Zimbabwe.

**Table 3:** Background characteristics of the sample by country and delivery location. Some percent distributions do not add up to 100% due to rounding

	Individual				Family						Context		Structural
	Age			Education	Family life	Wealth					Residence	Health services	Gender
	*< 20 years*	*20–34 years*	*35+ years*	*Secondary or higher education*	*Lives with husband*	*Poorest*	*Poorer*	*Middle*	*Richer*	*Richest*	*Urban*	*Community facility delivery rate*^a^	*Women’s social independence score* ^b^
	*n (%)*	*n (%)*	*n (%)*	*n (%)*	*n (%)*	*n (%)*	*n (%)*	*n (%)*	*n (%)*	*n (%)*	*n (%)*	*%*	
**Facility births**													
Angola	516 (20.7)	1676 (67.3)	299 (12.0)	1377 (55.3)	1675 (67.2)	141 (5.7)	328 (13.2)	629 (25.3)	698 (28.0)	695 (27.9)	2113 (84.8)	68.3	0.02
Benin	619 (13.5)	3351 (73.1)	614 (13.4)	964 (21.0)	3671 (80.1)	723 (15.8)	911 (19.9)	981 (21.4)	1012 (22.1)	956 (20.9)	1879 (41.0)	89.2	−0.15
Burundi	363 (7.9)	3402 (74.3)	811 (17.7)	613 (13.4)	3650 (79.8)	916 (20.0)	1007 (22.0)	947 (20.7)	871 (19.0)	835 (18.3)	457 (10.0)	86.5	0.06
Cameroon	479 (18.4)	1820 (69.9)	305 (11.7)	1503 (57.7)	1798 (69.1)	255 (9.8)	527 (20.2)	614 (23.6)	645 (24.8)	563 (21.6)	1494 (57.4)	82.5	0.06
Ethiopia	195 (12.8)	1141 (75)	185 (12.1)	317 (20.9)	1276 (83.9)	144 (9.4)	256 (16.8)	278 (18.3)	315 (20.7)	528 (34.7)	458 (30.1)	53.7	−0.23
Guinea	330 (20.5)	1047 (65.1)	231 (14.3)	350 (21.8)	1247 (77.6)	185 (11.5)	297 (18.5)	305 (19.0)	424 (26.4)	395 (24.6)	718 (44.7)	71.2	−0.37
Malawi	1278 (20.9)	4126 (67.5)	708 (11.6)	1355 (22.2)	4400 (72.0)	1501 (24.6)	1372 (22.5)	1174 (19.2)	1055 (17.3)	1010 (16.5)	867 (14.2)	92.6	−0.18
Mali	502 (17.6)	1894 (66.6)	450 (15.8)	634 (22.3)	2443 (85.8)	408 (14.3)	492 (17.3)	573 (20.1)	674 (23.7)	699 (24.6)	808 (28.4)	82.4	−0.39
Nepal	318 (25.2)	903 (71.7)	38 (3.0)	790 (62.7)	750 (59.6)	172 (13.7)	228 (18.1)	301 (23.9)	299 (23.8)	258 (20.5)	777 (61.7)	70.6	0.04
Nigeria	482 (9.5)	3819 (75.0)	793 (15.6)	3488 (68.5)	4261 (83.6)	340 (6.7)	649 (12.7)	1042 (20.5)	1411 (27.7)	1653 (32.5)	3011 (59.1)	67.2	0.31
Pakistan	239 (8.7)	2252 (81.9)	257 (9.4)	1280 (46.6)	2277 (82.8)	392 (14.3)	422 (15.3)	618 (22.5)	608 (22.1)	708 (25.8)	1078 (39.2)	74.9	0.28
Senegal	464 (13.1)	2477 (70.2)	590 (16.7)	771 (21.8)	2024 (57.3)	612 (17.3)	768 (21.7)	795 (22.5)	698 (19.8)	658 (18.6)	1483 (42)	84.6	0.02
Tanzania	553 (20.8)	1728 (65.1)	374 (14.1)	623 (23.5)	1956 (73.7)	439 (16.5)	455 (17.1)	472 (17.8)	627 (23.6)	662 (24.9)	992 (37.3)	75.6	0.14
Uganda	810 (18.3)	3102 (70.2)	509 (11.5)	1556 (35.2)	3101 (70.1)	855 (19.3)	840 (19.0)	815 (18.4)	835 (18.9)	1077 (24.4)	1106 (25.0)	79.3	−0.07
Zambia	722 (21.8)	2114 (63.7)	484 (14.6)	1443 (43.5)	2280 (68.7)	757 (22.8)	724 (21.8)	642 (19.3)	611 (18.4)	586 (17.7)	1237 (37.3)	87.9	−0.10
Zimbabwe	371 (18.9)	1381 (70.4)	209 (10.7)	1416 (72.2)	1318 (67.2)	417 (21.3)	374 (19.1)	348 (17.8)	485 (24.8)	336 (17.1)	638 (32.5)	81.2	0.22
**Home births**													
Angola	567 (20.4)	1792 (64.3)	426 (15.3)	377 (13.5)	1859 (66.7)	519 (18.6)	1013 (36.4)	932 (33.4)	231 (8.3)	91 (3.3)	1072 (38.5)	26.0	−0.34
Benin	91 (11.3)	591 (73.3)	124 (15.4)	24 (3.0)	695 (86.1)	133 (16.5)	412 (51.1)	199 (24.7)	56 (6.9)	7 (0.8)	192 (23.8)	52.2	−0.39
Burundi	36 (4.7)	552 (70.6)	193 (24.7)	30 (3.9)	623 (79.7)	158 (20.3)	267 (34.2)	178 (22.7)	139 (17.8)	39 (5.0)	26 (3.3)	70.3	0.02
Cameroon	266 (21.5)	813 (65.6)	160 (12.9)	151 (12.2)	1046 (84.5)	187 (15.1)	602 (48.7)	374 (30.2)	64 (5.2)	11 (0.9)	185 (14.9)	33.9	−0.52
Ethiopia	306 (11.3)	1924 (71.3)	470 (17.4)	53 (1.9)	2422 (89.7)	595 (22.0)	848 (31.4)	677 (25.1)	452 (16.7)	128 (4.7)	52 (1.9)	13.3	−0.52
Guinea	241 (18)	872 (65.1)	227 (16.9)	57 (4.3)	1139 (85.0)	270 (20.2)	510 (38.1)	373 (27.8)	123 (9.2)	64 (4.8)	128 (9.5)	30.0	−0.53
Malawi	82 (18)	286 (63)	87 (19.1)	40 (8.7)	310 (68.2)	93 (20.5)	162 (35.6)	120 (26.5)	53 (11.7)	26 (5.7)	32 (7.1)	76.8	−0.28
Mali	176 (14.3)	852 (69.3)	201 (16.4)	55 (4.5)	1071 (87.1)	290 (23.6)	413 (33.6)	390 (31.8)	104 (8.5)	32 (2.6)	50 (4.1)	35.2	−0.52
Nepal	127 (18.2)	536 (76.7)	36 (5.1)	223 (31.9)	413 (59.1)	150 (21.5)	238 (34.0)	181 (25.9)	105 (15.0)	26 (3.6)	277 (39.6)	36.4	−0.23
Nigeria	1119 (14.9)	5189 (69.0)	1214 (16.1)	1659 (22.1)	6854 (91.1)	1541 (20.5)	2382 (31.7)	2235 (29.7)	941 (12.5)	424 (5.6)	1851 (24.6)	20.9	−0.50
Pakistan	108 (9.8)	837 (75.7)	161 (14.6)	155 (14)	938 (84.8)	217 (19.6)	432 (39.0)	303 (27.4)	117 (10.6)	37 (3.3)	191 (17.3)	46.5	−0.05
Senegal	110 (12.6)	598 (68.7)	163 (18.7)	54 (6.2)	602 (69.1)	115 (13.2)	461 (53.0)	219 (25.2)	50 (5.8)	25 (2.8)	96 (11.0)	52.4	−0.36
Tanzania	212 (14.8)	963 (67.1)	260 (18.1)	72 (5.0)	1135 (79.1)	293 (20.4)	558 (38.9)	402 (28.0)	151 (10.5)	31 (2.2)	141 (9.8)	39.3	−0.14
Uganda	193 (14.2)	935 (68.8)	232 (17.1)	171 (12.6)	1028 (75.6)	281 (20.7)	439 (32.3)	385 (28.3)	181 (13.3)	74 (5.4)	130 (9.5)	54.8	−0.26
Zambia	95 (18.2)	323 (61.5)	107 (20.3)	91 (17.2)	381 (72.6)	82 (15.7)	234 (44.5)	139 (26.5)	45 (8.6)	24 (4.6)	77 (14.7)	58.4	−0.30
Zimbabwe	70 (15.3)	321 (70.3)	66 (14.4)	196 (42.9)	310 (68.0)	88 (19.3)	186 (40.8)	119 (26.1)	52 (11.5)	11 (2.3)	39 (8.6)	54.9	−0.09

^a^ Proportion of women in the community who had a birth in the last five years who gave birth in a health facility, calculated at the cluster level and applied to all women in the cluster

^b^ Survey-based Women’s emPowERment index (SWPER) [25] score calculated and averaged across all partnered women in each survey cluster and applied to all women in the cluster

The proportion of women living with their partners ranged from 57.3% in Senegal to 85.8% in Mali amongst facility births and from 59.1% in Nepal to 91.1% in Nigeria amongst home births. Among facility births the proportion in the richest wealth quintile ranged from 16.5% in Malawi to 32.5% in Nigeria. Among home births this ranged from 0.8% in Benin to 5.7% in Malawi. Urban residence was higher among facility births (10.0% in Burundi to 84.8% in Angola) than home births (3.3% in Burundi to 39.6% in Nepal). Community-level facility birth rates ranged from 53.7% in Ethiopia to 92.6% in Malawi amongst facility births and from 13.3% in Ethiopia to 76.8% in Malawi amongst home births. Cluster-level scores for women’s social independence were higher amongst facility births (− 0.39in Mali to 0.31 in Nigeria) than amongst home births (− 0.53 in Guinea to 0.02 in Burundi).

### Coverage of newborn care interventions

**Fig. 2:**
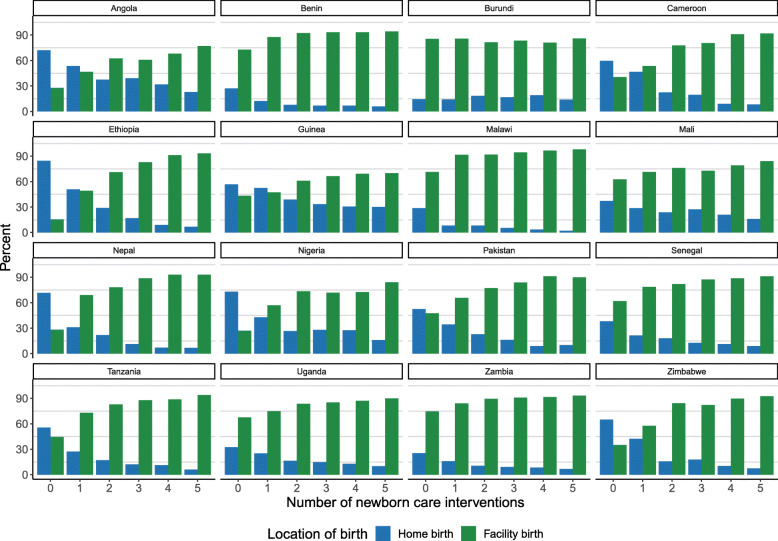
Proportion of facility or home birth for each level of co-coverage. This figure shows, for each level of co-coverage (0–5), the proportion of births that took place at home (shown in blue) and the proportion that took place in a health facility (shown in green)

Reported co-coverage of the five possible newborn care interventions considered varied between countries, lowest in Ethiopia and highest in Zimbabwe. Within countries, co-coverage varied widely based on place of birth in most countries in this study. Figure 2 shows the proportion of facility births increases as co-coverage increases in most countries. In Ethiopia, amongst newborns with no interventions (co-coverage = 0) 15.5% were born in a facility and 84.5% were born at home while amongst newborns with five interventions 93.2% were born in a facility. In Burundi, however, the proportion of facility versus home births was similar across all co-coverage levels (14.0–19.0% home births).

**Fig. 3:**
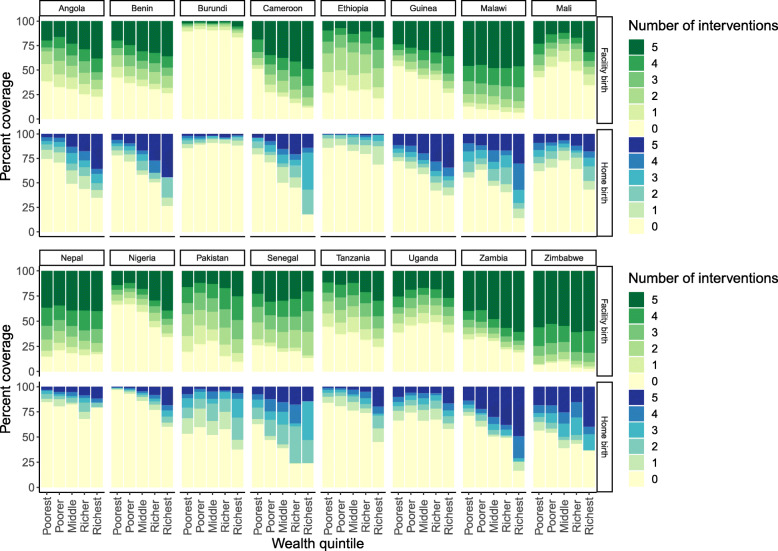
Percent distribution of co-coverage levels for each wealth quintile, by home and facility birth

Figure 3 shows the proportion of each co-coverage level for each wealth group, separately by location of birth. Among home births, wealth disparities can be seen in all but Burundi. Whilst less pronounced, wealth disparities persisted amongst facility births in most countries. In Benin, amongst facility births 19.5% of newborns in the poorest wealth quintile received all 5 interventions compared with 35.8% in the richest wealth quintile whilst amongst home births 5.8% of the poorest received all 5 interventions compared with 44.2% of the richest. In Zimbabwe, co-coverage is consistently high amongst all wealth groups for facility births (coverage of five interventions: 52.5–60.7%) with some wealth disparity amongst home births (18.0% of the poorest home births received 5 interventions compared with 39.6% of the richest). The relationship between co-coverage and wealth quintile was not linear in all countries. In Uganda, among facility births, co-coverage of 5 interventions in the poorest wealth quintile (25.3%) was more comparable to the richest quintile (26.7%) than the three middle quintiles (16.6–18.4%).

**Fig. 4:**
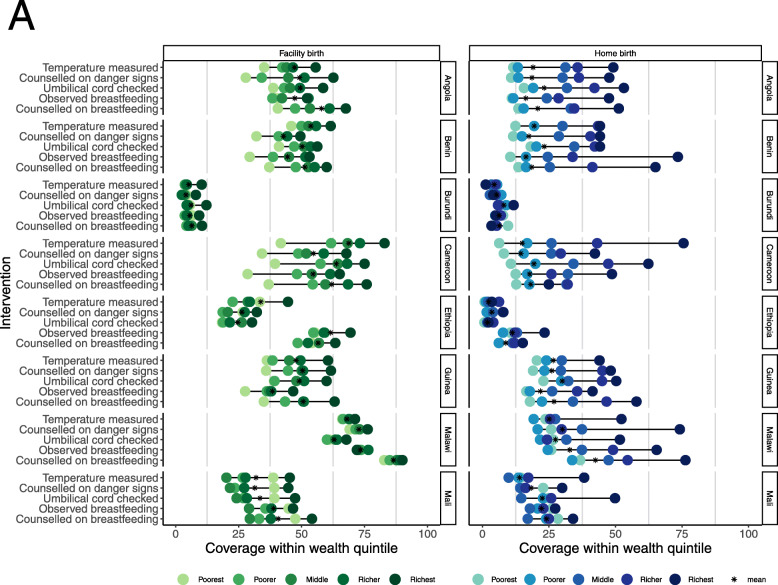
*(See caption on next page)*

Figure 4 shows coverage of each intervention within each wealth quintile, separately by location of birth. Similar to co-coverage, wealth inequities were more obvious among home births. Facility and home births in Angola showed a linear pattern of inequity across all interventions with wider disparities among home births. While a linear inequality pattern was seen across interventions among facility births in Tanzania and Malawi, a top-inequity pattern was seen in home births with the richest groups having 1.9–2.3 times higher coverage than the next wealth group in Tanzania and 1.3–2.5 times higher coverage in Malawi.

Bottom-inequity patterns are seen among facility births in Cameroon where despite birth in a facility, the poorest newborns receive the least care. Across the five interventions, newborns in the second wealth group had 1.3–2.6 times higher coverage than the poorest. Care was more equitable in some countries, for example, among both facility and home births in Uganda and Mali, the poorest wealth groups had higher coverage than the middle wealth groups. Among home births in Nepal, the poorest wealth groups had the highest coverage for some interventions, including counselling on danger signs (52.2% of newborns in the poorest group compared to 50.3% of the richest).

### Factors associated with increased co-coverage

Table 4 shows findings of multilevel logistic regression models for facility births each country, examining the relationship between appropriate care (reported co-coverage of 4-5 interventions) and individual, family, context, and structural factors. Crude associations are presented in Additional file 1.

**Table 4:** Results of multilevel, multivariable logistic regressions of newborn care co-coverage among facility births. Adjusted odds ratios and 95% confidence intervals are presented

	**Angola**	**Benin**	**Burundi**	**Cameroon**	**Ethiopia**	**Guinea**	**Malawi**	**Mali**
**Individual**								
Age (ref: < 20 years)								
20–24 years	0.94 (0.63–1.41)	0.87 (0.64–1.18)	0.77 (0.34–1.76)	1.04 (0.74–1.46)	1.83 (0.74–4.55)	0.66 (0.31–1.42)	1.00 (0.80–1.24)	0.91 (0.52–1.58)
35+ years	1.07 (0.58–1.97)	0.89 (0.58–1.35)	0.62 (0.24–1.60)	1.04 (0.68–1.60)	1.75 (0.51–6.05)	0.81 (0.32–2.04)	1.08 (0.81–1.45)	0.84 (0.46–1.53)
Secondary or higher education (ref: no education or primary only)	1.54 (1.00–2.37)	**1.38 (1.09–1.76)**	**1.86 (1.05–3.30)**	0.88 (0.68–1.14)	1.10 (0.53–2.29)	0.89 (0.45–1.79)	1.24 (0.99–1.55)	1.41 (0.98–2.03)
**Family**								
Lives with husband (ref: doesn’t live with husband)	0.81 (0.59–1.13)	1.06 (0.82–1.38)	**1.96 (1.01–3.82)**	**0.74 (0.57–0.96)**	1.11 (0.59–2.10)	1.05 (0.58–1.90)	0.96 (0.79–1.16)	0.65 (0.40–1.06)
Wealth (ref: poorest)								
Poorer	1.36 (0.53–3.50)	1.28 (0.90–1.83)	**0.42 (0.19–0.94)**	1.23 (0.73–2.10)	0.33 (0.10–1.06)	0.48 (0.14–1.63)	1.24 (0.97–1.57)	0.58 (0.27–1.25)
Middle	1.41 (0.48–4.18)	**1.72 (1.17–2.53)**	0.63 (0.25–1.60)	**2.05 (1.16–3.60)**	0.65 (0.17–2.42)	1.14 (0.40–3.30)	1.19 (0.93–1.53)	0.51 (0.26–1.00)
Richer	1.36 (0.47–3.91)	**1.74 (1.19–2.53)**	**0.26 (0.09–0.73)**	**2.50 (1.40–4.48)**	1.07 (0.28–4.01)	1.28 (0.40–4.09)	1.19 (0.91–1.56)	0.44 (0.17–1.12)
Richest	1.32 (0.42–4.18)	**1.89 (1.22–2.94)**	0.86 (0.36–2.04)	**2.49 (1.32–4.71)**	1.26 (0.28–5.75)	1.23 (0.31–4.86)	1.18 (0.82–1.70)	0.48 (0.17–1.36)
**Context**								
Urban (ref: rural)	1.15 (0.58–2.29)	1.02 (0.74–1.41)	**2.51 (1.03–6.14)**	0.92 (0.66–1.26)	1.49 (0.38–5.83)	0.98 (0.39–2.46)	1.30 (0.95–1.77)	1.93 (0.66–5.68)
Community facility delivery rate (ref: lowest)								
Middle	1.47 (0.59–3.68)	1.21 (0.81–1.79)	1.31 (0.58–2.97)	**2.40 (1.42–4.06)**	0.29 (0.06–1.39)	1.07 (0.42–2.70)	**1.47 (1.17–1.85)**	**0.31 (0.11–0.93)**
Highest	1.68 (0.66–4.29)	1.29 (0.42–4.00)	0.93 (0.40–2.18)	**2.61 (1.57–4.33)**	0.99 (0.22–4.47)	0.98 (0.38–2.56)	1.57 (0.53–4.66)	0.71 (0.23–2.15)
**Structural**								
Women’s social independence								
Middle	1.20 (0.62–2.32)	1.12 (0.75–1.66)	0.93 (0.42–2.06)	0.89 (0.62–1.30)	2.13 (0.83–5.45)	0.96 (0.43–2.14)	1.00 (0.78–1.28)	1.24 (0.43–3.63)
Highest	1.72 (0.89–3.31)	1.42 (0.94–2.14)	1.16 (0.53–2.51)	1.21 (0.84–1.76)	1.90 (0.77–4.66)	0.94 (0.43–2.08)	1.03 (0.78–1.36)	**3.94 (1.31–11.86)**
ICC (rho)	.4720	.3149	.5563	<.0001	.7933	.4968	.2657	.6590
Number of births	1915	3138	3796	1668	1639	712	5757	1659
Number of clusters	393	379	467	258	499	176	799	200
	**Nepal**	**Nigeria**	**Pakistan**	**Senegal**	**Tanzania**	**Uganda**	**Zambia**	**Zimbabwe**
**Individual**								
Age (ref: < 20 years)								
20–24 years	1.33 (0.72–2.47)	1.11 (0.79–1.56)	1.49 (0.59–3.79)	1.09 (0.81–1.46)	0.97 (0.65–1.46)	0.87 (0.67–1.13)	1.02 (0.72–1.45)	0.97 (0.55–1.70)
35+ years	1.75 (0.31–9.89)	1.16 (0.80–1.67)	2.58 (0.89–7.47)	**1.50 (1.01–2.22)**	0.75 (0.44–1.27)	1.09 (0.76–1.58)	0.62 (0.26–1.47)	1.64 (0.59–4.61)
Secondary or higher education (ref: no education or primary only)	1.23 (0.64–2.36)	1.10 (0.90–1.36)	1.56 (0.93–2.62)	**1.31 (1.02–1.68)**	**1.64 (1.10–2.44)**	**1.60 (1.26–2.04)**	1.22 (0.86–1.73)	1.30 (0.80–2.11)
**Family**								
Lives with husband (ref: doesn’t live with husband)	0.78 (0.49–1.26)	0.98 (0.77–1.24)	1.04 (0.64–1.69)	0.99 (0.79–1.26)	1.16 (0.79–1.70)	1.02 (0.80–1.29)	0.84 (0.60–1.16)	1.56 (0.98–2.49)
Wealth (ref: poorest)								
Poorer	0.80 (0.31–2.05)	0.70 (0.46–1.07)	1.06 (0.44–2.59)	1.43 (1.00–2.04)	1.11 (0.61–2.04)	0.75 (0.55–1.02)	1.00 (0.64–1.56)	1.08 (0.53–2.22)
Middle	0.99 (0.33–2.93)	0.76 (0.52–1.11)	1.82 (0.77–4.35)	**1.51 (1.00–2.28)**	1.02 (0.56–1.86)	0.76 (0.53–1.08)	1.41 (0.87–2.28)	0.97 (0.50–1.88)
Richer	1.42 (0.47–4.26)	1.04 (0.69–1.54)	2.23 (0.91–5.47)	1.73 (0.99–3.04)	0.98 (0.48–1.99)	0.81 (0.57–1.17)	1.51 (0.80–2.85)	1.72 (0.75–3.93)
Richest	0.94 (0.24–3.66)	1.25 (0.83–1.90)	**3.12 (1.21–8.09)**	1.12 (0.63–1.97)	0.96 (0.43–2.17)	1.16 (0.75–1.78)	1.97 (0.70–5.57)	1.62 (0.52–5.06)
**Context**								
Urban (ref: rural)	0.72 (0.37–1.38)	0.96 (0.79–1.17)	1.28 (0.84–1.96)	**0.71 (0.53–0.96)**	**2.26 (1.29–3.97)**	0.99 (0.65–1.50)	0.85 (0.52–1.41)	1.21 (0.42–3.44)
Community facility delivery rate (ref: lowest)								
Middle	0.83 (0.32–2.11)	**1.74 (0.97–3.13)**	**1.92 (1.06–3.46)**	1.11 (0.75–1.66)	**2.40 (1.21–4.77)**	0.86 (0.60–1.24)	0.96 (0.52–1.80)	0.93 (0.44–1.95)
Highest	0.74 (0.27–2.09)	**2.16 (1.18–3.98)**	**2.52 (1.35–4.71)**	1.04 (0.67–1.62)	**2.83 (1.38–5.82)**	0.84 (0.57–1.23)	1.33 (0.82–2.17)	0.82 (0.37–1.84)
**Structural**								
Women’s social independence								
Middle	2.04 (0.89–4.69)	1.16 (0.74–1.83)	0.73 (0.39–1.34)	**1.77 (1.21–2.58)**	1.51 (0.82–2.75)	1.06 (0.75–1.49)	1.21 (0.67–2.20)	1.65 (0.80–3.41)
Highest	**4.48 (1.80–11.18)**	**4.61 (2.88–7.40)**	0.58 (0.30–1.13)	**2.02 (1.35–3.02)**	**2.22 (1.12–4.40)**	0.82 (0.55–1.22)	0.76 (0.44–1.31)	1.12 (0.43–2.90)
ICC (rho)	.4682	.1888	.1192	.2377	.5694	.4353	.4061	.5301
Number of births	647	5071	1243	2891	2541	4439	1900	1348
Number of clusters	178	1194	210	316	554	689	325	261

#### Individual

Individual factors (age and education) were associated with appropriate newborn care (co-coverage of four or more) in five of the 16 countries in adjusted models. While maternal age at birth was significant only in Senegal (35+ years compared to < 20 years; AOR = 1.50, 95%CI = 1.01,2.22), secondary or higher education was a significant factor in Senegal (AOR = 1.31, 95%CI = 1.02,1.68) as well as Benin (AOR = 1.38, 95%CI = 1.09,1.76), Burundi (AOR = 1.86, 95%CI = 1.05,3.30),Tanzania (AOR = 1.64, 95%CI = 1.10,2.44), and Uganda (AOR = 1.60, 95%CI = 1.26,2.04).

#### Family

Living with a partner was positively associated with appropriate newborn care in Burundi (AOR = 1.96, 95%CI = 1.01,3.82), however negatively associated with appropriate newborn care in Cameroon (AOR = 0.74, 95%CI = 0.57,0.96).

While increasing wealth was associated with higher odds of appropriate newborn care in Benin, Cameroon, Pakistan, and Senegal, in Burundi, the poorer and richer wealth groups were associated with a decrease in the odds of appropriate care compared with the poorest group (Cameroon richer AOR = 2.50, 95%CI = 1.40,4.48; Burundi richer AOR = 0.26, 95%CI = 0.09,0.73; reference group is the poorest wealth quintile).

#### Context

Urban residence, as compared to rural, was associated with a large increase in the odds of appropriate care in Burundi (AOR = 2.51, 95%CI = 1.03,6.14) and Tanzania (AOR = 2.26, 95%CI = 1.29,3.97) but a 29% (AOR = 0.71, 95%CI = 0.53,0.96) decrease in the odds of appropriate newborn care in Senegal.

The community level facility delivery rate was associated with large increases in the odds of appropriate care in Cameroon, Malawi, Nigeria, Pakistan, and Tanzania, however, the middle tercile community facility delivery rate was associated with a 69% (AOR = 0.31, 95%CI = 0.11,0.93) decrease in the odds of appropriate newborn care as compared with the lowest tercile in Mali (Cameroon middle tercile AOR = 2.04, 95%CI = 1.42,4.06).

#### Structural

At the structural level, women’s social independence was associated with a significant increase in appropriate newborn care in Mali, Nepal, Nigeria, Senegal, and Tanzania (Nigeria highest tercile AOR = 4.61, 95%CI = 2.88,7.40, reference group is the lowest tercile).

None of the factors we assessed were significantly associated with appropriate newborn care in the adjusted models for Angola, Ethiopia, Guinea, Zambia or Zimbabwe.

## Discussion

This study found large between- and within-country gaps in reported co-coverage of newborn care for those born at home and in facilities. We found important inequities in appropriate newborn care based on specified individual, family, contextual, and structural factors. While we found large wealth-based differences in intervention coverage and co-coverage of newborn care, wealth was not a significant factor for co-coverage in all country models when controlling for other social determinants of health. This is an important finding because much of the existing research in inequities in coverage of maternal, newborn and child health has been focused on wealth-based inequities whilst the access to health care is known to be multi-dimensional [30]. Understanding, improving and sustaining equity in coverage is a key component of the WHO vision for maternal, newborn, and child health [3] and also essential to assessing and improving overall coverage of care [31] and improving newborn survival [4]. Priority strategic objectives for ending preventable newborn mortality include strengthening care around the time of birth and minimising inequities in access to and coverage of care [32].

Gaps in appropriate care for home births might be expected, as to receive provider-initiated interventions, the newborn would need to be transported to a facility, or a health care provider would need to travel to visit the newborn. In Malawi, facility birth is high and qualitative research has shown women will go to facilities the same day following home birth [33]. While we found consistently high coverage of interventions among facility births in Malawi, home births had wide wealth-based inequities. In other settings, cultural and religious practices may be a barrier to accessing care during the postnatal period where resting and seclusion are the norm [34]. Facility birth itself is distributed inequitably where 70% of births in the lowest two wealth quintiles occur at home in sub-Saharan Africa and south Asia [35] and both poverty and long travel time are barriers to hospital birth [5].

We also found gaps in appropriate care and wealth-based inequities in coverage for facility births, similar to a study of facility-births showing gaps in care, particularly for women with less education, and single women [36]. While facility birth and skilled attendance at birth are important steps to early newborn care, this alone may not lead to reduction in neonatal mortality [37]. Recent training on any aspect of essential childbirth care and availability of relevant guidelines at facilities in LMICs is limited and service availability, service readiness, and coverage of obstetric care services are low [38]. Even when a skilled provider is present at the delivery, much of the provider’s focus may be on the woman and not on the newborn; the provider’s capacity to deliver essential newborn care is not guaranteed [39]. Health system redesign to shift births to hospitals may improve coverage of appropriate newborn care in theory, however, requires significant political leadership and infrastructure and policy investment. To avoid exacerbating current disparities and improve equitable access to birth and newborn care, redesign will need to consider geographic, socioeconomic, and other barriers [40].

In a study of co-coverage of child-survival interventions, Victora et al. [20] found that low-coverage countries showed top inequity patterns (mass deprivation), while countries with increasing coverage showed linear inequity patterns, and high-coverage countries showed bottom inequity (marginalisation). We found this to be more consistent for home births where wealth-based inequities were much wider than facility births. Only Burundi and Ethiopia, where coverage was very low, did facility births have greater wealth-based inequities than home births. Similar to our findings of very low intervention coverage, even among facility births, a study of antenatal care showed most women in Burundi sought skilled antenatal care but very few received quality coverage (three interventions) [41]. This may be due to attempts to address equity through removal of user fees for pregnancy and newborn care which led to increased service utilisation but service-level challenges [42]. Research on maternal health services in Ethiopia has shown an increase in wealth-based inequities between 2000 and 2016 [43] with skilled birth attendance being the most inequitably distributed maternal health service [44]. Equitable coverage challenges in Ethiopia include geographical factors which intersect with poor infrastructure and limited subnational financial resources [45].

Uganda and Mali did not have a classic wealth inequity pattern where the poorest quintile of the population had coverage above the national mean for all interventions. Policies to address equity in healthcare provision may explain this finding though other countries with classic wealth inequity patterns have similar policies (e.g. Ethiopia). Uganda abolished user fees at public health centres and hospitals in 2001 and initiated a village health teams strategy to bridge the gap between health facilities and communities, a programme which was revised into a community health extension workers programme in 2016 [46]. Research has shown the poor have benefited from improved access to health services following removal of user fees; however, cost can still be a barrier to reach facilities or to quality care. Lack of commodities available at the facility leaves patients to purchase their own supplies; patients without the means to do so are further disadvantaged in receipt of quality services [47]. In Mali, a rural auxiliary midwife (“matrone”) programme has been in place since the 1970s with a scope of work including all aspects of reproductive health [48]. A systematic review of community health worker programmes showed that equity is best achieved when community health workers conduct home visits, facilitate community-based groups, and enable cash transfers as well as when they are well supported to assist families in decision making and to overcome barriers to access services [49].

We found inequities in co-coverage by wealth and education, similar to findings from a systematic review of inequities in use of postnatal services in LMICs [24]. While the systematic review found significantly higher coverage in urban areas as compared to rural [24], our regression models showed urban residence was significantly associated with increased co-coverage only in Senegal and Tanzania. Additionally, our study found women’s social independence was associated with increased co-coverage in six of the included countries. Studies of women’s empowerment and institutional delivery or skilled attendance at birth have shown decision-making power to be associated with improved coverage of intrapartum care in some countries [25, 50].

### Strengths and limitations

Much of the research in inequities around the time of birth focuses on a single intervention or contact with the health system. But many interventions and practices happen around birth so it is useful to consider a package of interventions. This study takes a range of newborn care interventions, creating a co-coverage measure to identify inequities in receipt of a broader suite of interventions. As a summary measure, co-coverage provides an important indicator of high-risk groups left behind in coverage of care. Furthermore, these measures can assess increased inequities from the packaging of several interventions [20].

Some limitations of the present study should be noted. Detailed analysis of the health and economic context within the countries included in this analysis was outside the scope of this study. Coverage measures were not adjusted for quality of the health interventions or health outcomes achieved [51]. Additionally, survey-based measurement of coverage of care is subject to the ability of respondents to understand the questions and accurately report the answers. Results from validation studies of some newborn care survey questions have shown inconsistent results [52–58]. In Kenya and Swaziland, for example, interventions such as counselling on breastfeeding and danger signs in the newborn met criteria for accuracy and bias while interventions such examining the baby did not [55]. To increase the likelihood of accurate recall, we limited the study population to last births in the 2 years before the survey.

The models to examine factors associated with appropriate newborn care were fit only for facility births as factors are likely to be different for those born in a facility and those born at home. With increasing facility births, tracking and addressing inequities in care amongst facility births is important but addressing gaps between home and facility births should not be neglected.

## Conclusion

Improving coverage and equity in newborn care is essential to improving newborn survival on a global scale. This study highlights important inequities both within and between countries for home and facility births. Community-based or pro-poor interventions are important to mitigate wealth-based inequities in access to birth and newborn care services, particularly in countries with large differences in access between the poorest and richest families such as Angola, Cameroon, and Zambia as well as in countries with very low coverage of care, such as Burundi and Ethiopia. Further research is needed to understand access to quality newborn care across additional social determinants of health and in localized contexts.

## Supplementary Information

**Additional file 1.** Crude odds ratios for factors associated with newborn care co-coverage, by country.

## Data Availability

The dataset was compiled from data provided by the DHS program. Data are available for use upon registration: https://www.dhsprogram.com/Data/
