# Supplementary material for: Equity in newborn care, evidence from national surveys in low- and middle-income countries
Source: Int J Equity Health. 2021 Jun 5;20:132. doi: 10.1186/s12939-021-01452-z (PMC8178885; doi:10.1186/s12939-021-01452-z)
Supplement: Supplementary file 1 — Additional file 1. Crude odds ratios for factors associated with newborn care co-coverage, by country. [file 12939_2021_1452_MOESM1_ESM.pdf]

Equity in newborn care, evidence from national surveys in low- and middle-income countries

Additional File 1

Crude odds ratios for factors associated with newborn care co-coverage, by country

	**Angola**	**Benin**	**Burundi**	**Cameroon**	**Ethiopia**	**Guinea**	**Malawi**	**Mali**
**Individual**								
Age (ref: <20 years)								
20-24 years	0.89 (0.60, 1.32)	0.85 (0.63, 1.16)	0.90 (0.39, 2.09)	1.21 (0.89, 1.65)	2.00 (0.80, 4.99)	0.69 (0.34, 1.43)	1.01 (0.81, 1.25)	0.85 (0.49, 1.48)
35+ years	0.89 (0.49, 1.59)	0.82 (0.55, 1.22)	0.74 (0.28, 1.92)	1.23 (0.81, 1.86)	1.89 (0.62, 5.72)	0.85 (0.33, 2.19)	1.06 (0.80, 1.42)	0.75 (0.42, 1.34)
Secondary or higher education (ref: no education or primary only)	**1.62 (1.10, 2.37)**	**1.51 (1.21, 1.89)**	**2.17 (1.19, 3.95)**	**1.48 (1.16, 1.87)**	1.25 (0.58, 2.68)	0.96 (0.50, 1.82)	**1.33 (1.08, 1.63)**	**1.43 (1.01, 2.01)**
**Family**								
Lives with husband (ref:	0.82 (0.60, 1.12)	1.02 (0.79, 1.32)	1.59 (0.85, 2.98)	**0.68 (0.53, 0.88)**	1.31 (0.66, 2.57)	1.06 (0.59, 1.89)	0.97 (0.80, 1.16)	0.61 (0.37, 1.02)

doesn't live with husband)								
Wealth (ref: poorest)								
Poorer	1.55 (0.64, 3.72)	1.33 (0.93, 1.89)	0.45 (0.21, 0.98)	**2.24 (1.39, 3.64)**	0.32 (0.10, 1.05)	0.49 (0.14, 1.68)	1.24 (0.98, 1.56)	0.60 (0.28, 1.28)
Middle	1.85 (0.76, 4.48)	**1.82 (1.25, 2.66)**	0.71 (0.29, 1.75)	**3.88 (2.35, 6.40)**	0.66 (0.18, 2.46)	1.11 (0.38, 3.21)	1.22 (0.95, 1.56)	0.55 (0.28, 1.07)
Richer	2.02 (0.85, 4.83)	**1.93 (1.34, 2.77)**	0.31 (0.11, 0.84)	**4.88 (2.98, 7.98)**	1.13 (0.30, 4.19)	1.27 (0.43, 3.75)	1.29 (0.99, 1.68)	0.59 (0.26, 1.36)
Richest	2.15 (0.86, 5.39)	**2.27 (1.52, 3.41)**	1.58 (0.76, 3.29)	**5.04 (3.03, 8.38)**	1.43 (0.32, 6.34)	1.17 (0.35, 3.92)	**1.48 (1.10, 1.99)**	0.77 (0.30, 1.94)
**Context**								
Urban (ref: rural)	**2.13 (1.26, 3.59)**	**1.32 (1.01, 1.72)**	**4.27 (2.21, 8.24)**	**1.75 (1.34, 2.28)**	**5.49 (2.65, 11.36)**	1.28 (0.68, 2.40)	**1.60 (1.28, 2.00)**	**3.09 (1.45, 6.56)**
Community facility delivery rate (ref: lowest)								
Middle	1.82 (0.80 - 4.16)	1.74 (1.22 - 2.49)	1.57 (0.66 - 3.72)	**3.81 (2.39 - 6.06)**	**0.49 (0.11 - 2.18)**	1.11 (0.44 - 2.78)	**1.58 (1.27 - 1.96)**	**0.26 (0.09 - 0.75)**
Highest	**3.06 (1.38 - 6.78)**	1.82 (0.62 - 5.32)	1.95 (0.88 - 4.31)	**4.74 (3.14 - 7.16)**	**2.86 (0.72 - 11.29)**	1.16 (0.52 - 2.58)	1.52 (0.51 - 4.49)	1.10 (0.40 - 3.01)
**Structural**								
Women's social independence								
Middle	1.39 (0.75 - 2.59)	1.23 (0.84 - 1.81)	1.03 (0.46 - 2.32)	**1.65 (1.07 - 2.54)**	**2.51 (1.02 - 6.16)**	0.95 (0.43 - 2.12)	1.08 (0.84 - 1.38)	1.63 (0.58 - 4.57)

Highest	**2.48 (1.38 - 4.45)**	**1.91 (1.33 - 2.73)**	1.88 (0.91 - 3.90)	**2.76 (1.92 - 3.98)**	**5.30 (2.36 - 11.91)**	0.97 (0.44 - 2.11)	**1.33 (1.04 - 1.70)**	**6.83 (2.47 - 18.92)**
Observations	1,915	3,138	3,796	1,668	1,639	712	5,757	1,659
Number of clusters	393	379	467	258	499	176	799	200
	**Nepal**	**Nigeria**	**Pakistan**	**Senegal**	**Tanzania**	**Uganda**	**Zambia**	**Zimbabwe**
**Individual**								
Age (ref: <20 years)								
20-24 years	1.28 (0.71, 2.33)	1.24 (0.79, 1.95)	1.80 (0.75, 4.34)	1.06 (0.79, 1.42)	1.04 (0.70, 1.55)	0.93 (0.72, 1.19)	0.97 (0.69, 1.36)	1.03 (0.59, 1.80)
35+ years	1.49 (0.30, 7.46)	1.07 (0.65, 1.75)	2.67 (0.93, 7.67)	1.41 (0.95, 2.09)	0.74 (0.44, 1.25)	1.07 (0.74, 1.54)	0.54 (0.22, 1.33)	1.69 (0.61, 4.65)
Secondary or higher education (ref: no education or primary only)	1.24 (0.68, 2.24)	1.28 (0.96, 1.71)	**2.42 (1.58, 3.69)**	1.22 (0.95, 1.57)	**1.70 (1.18, 2.45)**	**1.59 (1.25, 2.01)**	**1.49 (1.08, 2.04)**	1.31 (0.81, 2.12)
**Family**								
Lives with husband (ref:	0.80 (0.50, 1.29)	1.01 (0.74, 1.39)	1.02 (0.63, 1.67)	1.00 (0.79, 1.26)	1.08 (0.74, 1.57)	1.02 (0.80, 1.29)	0.75 (0.52, 1.09)	1.53 (0.96, 2.45)

doesn't live with husband)								
Wealth (ref: poorest)								
Poorer	0.90 (0.36, 2.24)	0.97 (0.50, 1.90)	1.20 (0.48, 3.00)	**1.51 (1.07, 2.13)**	1.12 (0.62, 2.04)	0.78 (0.58, 1.06)	1.01 (0.66, 1.56)	1.14 (0.55, 2.37)
Middle	1.19 (0.42, 3.33)	1.16 (0.60, 2.23)	**2.41 (1.09, 5.34)**	**1.66 (1.14, 2.42)**	1.11 (0.61, 2.00)	0.79 (0.56, 1.13)	1.47 (0.94, 2.29)	0.98 (0.51, 1.87)
Richer	1.69 (0.61, 4.70)	1.47 (0.73, 2.97)	**3.57 (1.63, 7.82)**	**1.98 (1.23, 3.17)**	1.27 (0.66, 2.44)	0.91 (0.64, 1.29)	1.57 (0.89, 2.77)	1.85 (0.88, 3.89)
Richest	1.16 (0.34, 3.99)	1.36 (0.64, 2.87)	**5.69 (2.63, 12.31)**	1.34 (0.84, 2.14)	1.63 (0.82, 3.25)	1.36 (0.92, 2.02)	2.23 (0.82, 6.08)	1.91 (0.72, 5.08)
**Context**								
Urban (ref: rural)	0.95 (0.53, 1.70)	**3.65 (2.82, 4.72)**	**2.33 (1.54, 3.54)**	1.07 (0.85, 1.35)	**4.13 (2.73, 6.24)**	1.18 (0.84, 1.66)	1.18 (0.79, 1.76)	**1.94 (1.12, 3.34)**
Community facility delivery rate (ref: lowest)								
Middle	1.08 (0.43 - 2.68)	**3.10 (1.72 - 5.56)**	**2.37 (1.32 - 4.27)**	1.40 (0.95 - 2.08)	**3.27 (1.64 - 6.54)**	0.90 (0.63 - 1.30)	1.03 (0.58 - 1.84)	1.07 (0.52 - 2.17)
Highest	1.35 (0.59 - 3.13)	**10.42 (6.01 - 18.08)**	**4.20 (2.32 - 7.60)**	**1.58 (1.12 - 2.24)**	**6.98 (3.72 - 13.10)**	0.97 (0.68 - 1.38)	1.42 (0.91 - 2.21)	1.20 (0.59 - 2.44)
**Structural**								
Women's social independence								
Middle	1.92 (0.88 - 4.17)	**1.62 (1.06 - 2.47)**	1.27 (0.64 - 2.54)	**1.98 (1.38 - 2.84)**	**2.04 (1.13 - 3.71)**	1.09 (0.78 - 1.53)	1.38 (0.80 - 2.37)	1.85 (0.94 - 3.65)

Highest	**4.12 (1.86 - 9.11)**	**8.83 (6.03 - 12.93)**	2.49 (1.35 - 4.58)	**2.16 (1.59 - 2.94)**	**5.18 (2.91 - 9.23)**	1.02 (0.73 - 1.43)	1.04 (0.64 - 1.70)	1.76 (0.89 - 3.47)
Observations	647	5,071	1,243	2,891	2,541	4,439	1,900	1,348
Number of clusters	178	1,194	210	316	554	689	325	261
